# Bridging the Gap: Animal Models in Next-Generation Reproductive Technologies for Male Fertility Preservation

**DOI:** 10.3390/life14010017

**Published:** 2023-12-21

**Authors:** Pedro M. Aponte, Miguel A. Gutierrez-Reinoso, Manuel Garcia-Herreros

**Affiliations:** 1 Colegio de Ciencias Biológicas y Ambientales (COCIBA), Universidad San Francisco de Quito (USFQ), Quito 170901, Ecuador; 2 Instituto de Investigaciones en Biomedicina “One-Health”, Universidad San Francisco de Quito (USFQ), Campus Cumbayá, Quito 170901, Ecuador; 3 Facultad de Ciencias Agropecuarias y Recursos Naturales, Carrera de Medicina Veterinaria, Universidad Técnica de Cotopaxi (UTC), Latacunga 050150, Ecuador; miguel.gutierrez@utc.edu.ec; 4 Laboratorio de Biotecnología Animal, Departamento de Ciencia Animal, Facultad de Ciencias Veterinarias, Universidad de Concepción (UdeC), Chillán 3780000, Chile; 5 Instituto Nacional de Investigação Agrária e Veterinária (INIAV), 2005-048 Santarém, Portugal

**Keywords:** animal models, male infertility, spermatogenesis, spermatogonial stem cells (SSCs), reproductive technologies, in vitro spermatogenesis, testicular tissue culture, cryopreservation, germ cell culture, transplantation

## Abstract

This review aims to explore advanced reproductive technologies for male fertility preservation, underscoring the essential role that animal models have played in shaping these techniques through historical contexts and into modern applications. Rising infertility concerns have become more prevalent in human populations recently. The surge in male fertility issues has prompted advanced reproductive technologies, with animal models playing a pivotal role in their evolution. Historically, animal models have aided our understanding in the field, from early reproductive basic research to developing techniques like artificial insemination, multiple ovulation, and in vitro fertilization. The contemporary landscape of male fertility preservation encompasses techniques such as sperm cryopreservation, testicular sperm extraction, and intracytoplasmic sperm injection, among others. The relevance of animal models will undoubtedly bridge the gap between traditional methods and revolutionary next-generation reproductive techniques, fortifying our collective efforts in enhancing male fertility preservation strategies. While we possess extensive knowledge about spermatogenesis and its regulation, largely thanks to insights from animal models that paved the way for human infertility treatments, a pressing need remains to further understand specific infertility issues unique to humans. The primary aim of this review is to provide a comprehensive analysis of how animal models have influenced the development and refinement of advanced reproductive technologies for male fertility preservation, and to assess their future potential in bridging the gap between current practices and cutting-edge fertility techniques, particularly in addressing unique human male factor infertility.

## 1. Introduction

Over the past few decades, the domain of reproductive biomedicine has been at the forefront of profound technological advancements, highlighting the escalating imperative to preserve male fertility. As the global incidence of male infertility continues to rise due to many factors—from lifestyle changes and environmental factors to genetic predispositions—there is an augmented interest in innovative methods to mitigate these challenges. Historically, most of these advancements have been made possible through the extensive use of animal models, allowing researchers to explore, test, and refine techniques in a controlled setting before their application to human subjects. This review highlights the integral role of animal models as the cornerstone of investigations in emerging reproductive technologies for male fertility preservation, bridging the gap between laboratory innovation and clinical implementation for the treatment of male factor infertility.

### 1.1. Overview of Male Fertility Issues

Human male fertility has witnessed a concerning decline over recent decades, with various studies indicating a notable decrease in sperm counts, motility, and overall male reproductive health. This reduction in male fertility is multifaceted, encompassing a wide array of physiological and environmental causes. Physiological challenges include genetic abnormalities, hormonal imbalances, and various health conditions like varicocele or infections affecting the reproductive tract. At the genetic level, anomalies like Y-chromosome microdeletions or mutations in specific genes can lead to sperm production issues. Hormonal imbalances, on the other hand, can affect the entire gametogenesis process, leading to reduced sperm production or compromised sperm health.

Beyond these physiological challenges, environmental and lifestyle factors have significantly contributed to the declining male fertility trend. Exposures to endocrine-disrupting chemicals (EDCs), commonly found in plastics, pesticides, and industrial chemicals, can interfere with hormonal pathways crucial for sperm production and maturation. Additionally, lifestyle elements such as poor diet, smoking, excessive alcohol consumption, obesity, and even psychological stress have been associated with adverse effects on sperm quality and overall male reproductive capability. As the global community grapples with these diverse challenges, the quest for effective and innovative fertility preservation techniques becomes even more paramount.

#### Classification of Human Infertility Conditions

Causes of male factor infertility can be divided into pre-testicular, testicular, and post-testicular factors. Other specific groups of conditions include immunological, environmental, and idiopathic. Pre-testicular factors refer to conditions or influences outside of the testicles that can impact sperm production or function. These can include the following: testicular factors, which pertain to conditions or issues directly related to the testes that affect their ability to produce healthy sperm; and post-testicular factors, which refer to conditions or obstructions that affect the transport or delivery of sperm post-production in the testes. These can include issues with parts of the male reproductive tract that transport, store, or protect sperm, such as the efferent ducts, epididymis, vas deferens, male glands, and urethra.

Immunological factors usually include the production of auto-anti-sperm Antibodies. Some men produce antibodies that attack their sperm, harming their fertility [1].

Environmental and lifestyle factors contribute to some infertility conditions, while other causes are unknown (idiopathic). For comprehensive details about infertility causes in men, see Table 1.

### 1.2. Animal Models and Male Infertility Overview

Animal models have been pivotal in understanding the fundamental aspects of male reproduction and developing and refining novel interventions for fertility preservation.

Techniques such as intracytoplasmic sperm injection (ICSI) and in vitro fertilization (IVF) were initially honed in animals. With their fast breeding and well-understood reproductive biology, rodents have been particularly instrumental, while larger animals like rabbits and non-human primates offer a closer physiological match to humans. These models are not only crucial for genetic studies, including gene knockout and overexpression techniques, but also for examining the impact of environmental and lifestyle factors on fertility. Recent use of animal models extends to assessing the safety and efficacy of new technologies like mitochondrial replacement therapy and gene editing. Beyond research, they are invaluable for training in various reproductive procedures. Despite their critical role, it is essential to acknowledge their limitations, as they do not fully replicate human biology, necessitating careful interpretation of results derived from these models. Table 2 presents examples and a summary of the advantages and limitations of some of the most used animal models in fertility studies and advancements.

## 2. Historical Aspects of Animal Models for Reproductive Technologies

**Figure 1:**
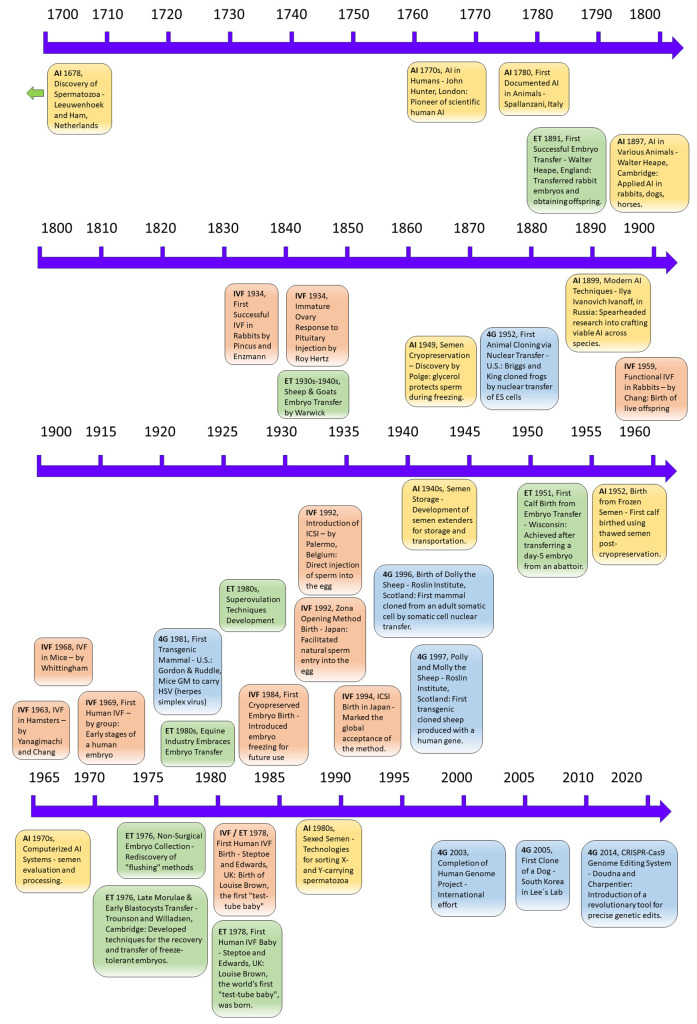
Evolution of Reproductive Technologies: A Historical Perspective. This figure illustrates the progression of four generations of reproductive biotechnologies, each building upon the foundations of the previous.

The progression of reproductive technologies in animal research has been instrumental in advancing human infertility treatments, with discoveries in animals marking four distinct generations of biotechnologies [35]. The first generation is artificial insemination (AI), which involves collecting and storing semen from male animals and the subsequent artificial deposition of this semen into the reproductive tract of female animals. AI has been widely implemented worldwide and extended to humans and various domestic species [36]. The second generation is the transfer of embryos collected in vivo (embryo transfer, ET). This technique involves the collection of embryos from a donor female animal and their transfer into the reproductive tract of recipient female animals. ET allows for overcoming infertility conditions in human couples [37]. The third generation is in vitro fertilization (IVF). This technique involves the fertilization of oocytes (eggs) outside of the female animal’s body in a laboratory setting. IVF allows for the manipulation and control of the fertilization process, and the resulting embryos can be cultured in vitro until they reach a stage suitable for transfer [38]. The fourth generation of reproductive biotechnologies in animal reproduction includes cloning by nuclear transfer and transgenesis [39,40]. Each generation of reproductive biotechnology has built upon the previous one. AI laid the foundation for the subsequent development of ET and IVF. IVF further expanded the possibilities for genetic manipulation and allowed for the production of embryos outside of the female animal’s body. These reproductive biotechnologies have shown remarkable adaptability and have been successfully transferred from research laboratories to farms worldwide [35]. However, their usefulness extends beyond the realm of animal breeding. These biotechnologies have revolutionized the study and treatment of human infertility. By applying the knowledge and techniques developed in animal reproduction, scientists and medical professionals can explore new avenues for understanding and addressing human reproductive challenges. A comprehensive summary of historical milestones of reproductive biotechnologies is presented in Figure 1.

**(1) Artificial Insemination (AI):** The collection, storage, and deposition of semen into the female’s reproductive tract. Antoni van Leeuwenhoek and his assistant Johannes Ham discovered spermatozoa in the Netherlands in 1678 [36]. Italian physiologist Lazzaro Spallanzani performed the first documented AI in animals in 1780, demonstrating that it was possible to use semen from one animal to impregnate another [41,42,43]. John Hunter, a Scottish surgeon, conducted during the 1770s the first documented application of AI in humans in London. He is considered the founder of scientific human AI [36]. In the 19th century (1897), Walter Heape, a reproductive biologist from Cambridge, reported using AI in rabbits, dogs, and horses [36]. In 1899, Ilya Ivanovich Ivanoff, a Russian researcher born in 1870, initiated efforts to craft viable techniques for artificial insemination. His investigations encompassed a range of species: farm animals, dogs, rabbits, and poultry. He led the way in using top-quality stallions to increase offspring through AI. Subsequently, another Russian scientist, Milovanov, built upon Ivanoff’s research introducing key cattle breeding initiatives and designed the earliest artificial vaginas, which resemble the current models [36]. In the early 20th century, AI was first successfully applied to cattle in Russia and Denmark [44]. The first large-scale bovine AI organization was established in Denmark in 1936, followed by similar organizations worldwide [42,44,45]. The development of semen extenders, which allowed semen to be stored and transported, was a major advancement in the 1940s [41]. The discovery in 1949 by Dr. Christopher Polge that glycerol protected sperm during cryopreservation revolutionized the field [46]. The first calf was born after insemination with frozen-thawed semen in 1952 [47]. In the 1970s, the introduction of computerized systems for semen evaluation and processing improved the efficiency and accuracy of AI [44]. Developing technologies to dependably sort X- and Y-carrying spermatozoa in the 1980s was a major milestone, and sexed semen for cattle became commercially available in the 1990s [41] [20]. With the advent of in vitro fertilization (IVF) in the 1970s, semen preparation techniques were refined. These techniques involved the removal of prostaglandins, infectious agents, and antigenic proteins from the semen, as well as isolating and selecting sperm cells with intact functional and genetic properties [36], but importantly, the rise of interest in artificial insemination for humans. Originally, unprocessed ejaculates were used for intrauterine insemination, leading to discomfort and the risk of infections. However, with IVF’s introduction, improved semen preparation techniques emerged, making intrauterine insemination safer and more relaxed [36]. **(2) Embryo Transfer (ET):** Collection of embryos from donor females and their transfer to recipient females, i.e., Embryo Transfer (ET). This technology, originally developed in animals, has proven to be a valuable tool for studying and addressing issues related to human male infertility. ET involves the transfer of embryos from a donor to a recipient animal, allowing researchers to explore fundamental aspects of reproduction, genetics, and embryology in various animal models and shedding light on the mechanisms underlying human male infertility. In 1891, Walter Heape successfully transferred two fertilized ova (embryos) from an Angora doe rabbit into the uterine tube of a Belgian Hare recipient [48], obtaining offspring. This early experiment demonstrated the feasibility of ET and hinted at the complexities associated with embryo implantation. ET was then extended to other farm animals, with successful procedures performed in sheep and goats in the 1930s–1940s by Warwick and colleagues [49]. Umbaugh (1949) was the first to report successful ET in cattle, resulting in four pregnancies. By 1951, the first calf from an embryo transfer was born in Wisconsin after the surgical transfer of a day-5 embryo obtained from an abattoir [49]. In the 1970s, while ET became commercially viable primarily through surgical methods, it was by 1976 that non-surgical collection methods were rediscovered and integrated into research [48]. In 1976, several groups reported an efficient, nontraumatic, nonsurgical technique using Foley catheters and the “flushing” method. British scientists Patrick Steptoe and Robert Edwards carried out the first successful human ET from in vitro fertilization that resulted in a live birth [50] of Louise Brown on July 25, 1978, the world’s first “test-tube baby,”. Robert Edwards was awarded the Nobel Prize in Physiology or Medicine in 2010 for this achievement. Superovulation techniques were developed in the 1980s [51]. **(3) In vitro Fertilization (IVF):** Fertilization of oocytes in a laboratory setting, allowing for external embryo cultivation. In the late 19th century, Walter Heape (UK) transferred fertilized ova from an Angora doe rabbit into the Fallopian tube of a Belgian hare recipient, resulting in the birth of six young, two of which displayed Angora phenotypes [52]. This first recorded embryo transfer opened the door for the possibility of fertilization outside the body, which was indeed achieved in 1934 when Pincus and Enzmann reported the first successful IVF in rabbits [52,53,54]. In the same year, Roy Hertz observed the response of immature rabbit ovaries to the injection of pituitary substances [55]. In 1959, Dr. Min Chueh Chang achieved the first successful and functional IVF procedure in rabbits [56,57,58]. In 1963, IVF was successfully achieved in hamsters [59] and by 1968, in mice [60]. Robert Edwards, Barry Bavister, and Patrick Steptoe published the first convincing evidence of the early stages of an embryo from human egg fertilization in vitro in 1969 [61]. In 1978, the world’s first human IVF pregnancy and birth of Louise Brown occurred in Oldham, UK [53,62]. During the early 1980s, cryopreservation was introduced, allowing the freezing and storage of embryos for future use. Introduced in 1983, the first baby’s birth from a cryopreserved embryo was born [63]. In 1985, a pregnancy was achieved using sperm aspirated from the epididymis [64] and culture media (Human Tubal Fluid) simulating the in vivo environment for embryos was introduced [65]. In 1992, intracytoplasmic sperm injection (ICSI) was introduced by Dr. Gianpiero Palermo and his team in Belgium with the generation of human offspring [66]. In Japan, the first baby was born through microinsemination using the zona opening method. This technique facilitates sperm’s natural entry into the egg by creating an opening in the zona pellucida. In 1990, the first successful birth following preimplantation genetic testing (PGT) was achieved [52]. **(4) Fourth generation of reproductive technologies—diploid cells (4G), Cloning and Transgenesis:** Advanced techniques involving nuclear transfer and genetic modification. These technologies, originating from animal reproduction research, have been pivotal in revolutionizing the study and treatment of human infertility through the use of animal models and are under current development.

## 3. Current State of Male Fertility Preservation (Sperm and Tissue Cryopreservation)

Over the past few decades, strides in reproductive medicine have brought to light the profound significance of male fertility preservation. This section unravels the intricacies of commonly used techniques, highlighting their application, efficiency, and the challenges they present. Male fertility preservation is essential for medical treatments affecting gonadal tissues, combining practical science with innovative approaches.

### 3.1. Commonly Used Techniques in Fertility Preservation

#### 3.1.1. Sperm Cryopreservation

Male fertility preservation generally focuses attention on sperm cryopreservation as the most common technique in animals and humans [67,68]. This method is standard in the medical field before chemotherapeutic treatments, which are toxic to gonadal tissues [69]. Moreover, it has also been used in autoimmune disease cases, avoiding the detrimental effects of the disease and treatments on germinal cells and tissues [70].

In farm animals, sperm cryopreservation has been routinely used to produce artificial insemination doses, improving offspring characteristics in breeding programs applied to the livestock industry [71], except for swine pig sperm freezing, which is still very challenging, and cool-stored (15–19 °C) semen is routinely used in pig artificial insemination worldwide [72,73]. The scientific community perceives that semen freezing, storage, and use for artificial insemination are safe, with pregnancy rates using semen from fertile individuals comparable to that after natural conception [74]. Sperm cryopreservation is a well-proven methodology for both farm animal species and humans [75].

Moreover, sperm cryopreservation protocols have been developed in several fish species but are challenging to reproduce in others [76]. The zebrafish (*Danio rerio*) has become a fish model for improving reproduction in other marine and freshwater commercial species [77]. Nowadays, there are several approaches to generating male gametes, such as testicular tissue, stem cell transplantation, and in vitro spermatogenesis. However, sperm cryopreservation protocols remain the most frequently used technique for preserving whole zebrafish gonads and germ cells [78]. In the near future, the preservation of immature cells (e.g., spermatogonial cryopreservation) and tissues will be helpful if proper maturation and differentiation techniques are applied after the cryopreservation process to produce fertile germ cells. Breeding techniques such as gamete handling, in vitro fertilization, intracytoplasmic sperm injection, and androgenesis have received more attention in recent years [79]. Somatic and sperm cryopreservation is already possible and is a reliable tool for storing genetic material for fertility preservation.

#### 3.1.2. Sperm Collection for Cryopreservation and ARTs

Sperm collection is a crucial step in the cryopreservation protocol because, depending on the procedure and the species, it will guarantee good-quality cryopreserved sperm samples [80]. In murine, usually, sperm collection is performed by killing the animal [81]. However, the sperm obtained using this method comes from the cauda of the epididymis, which is not a whole ejaculate sample (semen) [82]. Therefore, techniques have been developed for ejaculated sperm collection in several mammalian models. Electroejaculation was optimized for mice by studying the device type, waveform type of stimulus, probe type, and anesthetic compound [83]. Sperm collection from the female reproductive tract is another feasible approach [84]. In another rodent, the rat, penile vibratory devices exist [85] besides epididymal aspiration [86], while in hamsters, sperm is typically collected by sacrifice and *cauda epididymis* extraction [87].

Semen collections in rabbits are performed through artificial vaginas [88], and more recently, a reproducible and inexpensive optimized such device has been developed [89].

Artificial vagina and electroejaculation are the commonly used semen collection methods in ruminants [90,91]; and in horses, the most common practice is the use of an artificial vagina [92,93], while electroejaculation and epididymal extraction are less used [94].

In pigs [95], a gloved-handed technique is used to collect semen, while in dogs, besides gloved handed, there are artificial vaginas available [96].

Many sperm collection techniques and cryopreservation protocols have been developed during the last years to preserve sperm cells from non-human primate species with satisfactory results [97]. Non-human primates stand out as the group of species for which a remarkably varied array of semen collection techniques has been developed, underscoring their close physiological and genetic similarity to humans. Techniques range from electro-ejaculation and penile vibratory stimulation to more intricate approaches like retrieving sperm directly from the female reproductive tract or employing microsurgery for epididymal sperm collection [98]. Most sperm cryopreserved samples from non-human primates and other mammalian species have been used for IVF, ICSI and AI [99].

The fact that semen collection techniques are widespread across diverse animal models has served as a critical bridge to advancements in cryopreservation techniques and, consequently, the treatment of male factor infertility. These insights can lead to more effective strategies for preserving human sperm, especially when conventional cryopreservation methods face limitations. In oligospermia, characterized by a very low sperm count, there are insufficient sperm to freeze effectively, which poses a challenge for cryopreservation [100]. Cancer patients, particularly those undergoing treatments like chemotherapy or radiation, may not have the opportunity to produce multiple samples for cryopreservation, or their sperm quality might be compromised due to the illness [100]. When few sperm or sperm parameters like motility or morphology have deviated from normal, single-sperm cryopreservation can be a suitable option [101]. This technique involves protecting single sperm in a carrier, such as an empty zona pellucida, a technique developed using pre-fertilization mouse and hamster oocytes [102].

In summary, insights gained from successful semen collection and preservation in animal models can inform and enhance the quality and reliability of sperm banking for individuals facing medical treatments or fertility challenges.

#### 3.1.3. Testicular Tissue Cryopreservation

Testicular tissue is considered an alternative option for male fertility preservation, mainly when sperm cryopreservation is not feasible [103]. Cryopreservation of human testicular tissue is particularly beneficial in clinical scenarios such as cancer treatment, where future fertility may be compromised. This technique is also crucial in cases of cryptorchidism, a condition where one or both testes fail to descend, affecting the development and subsequent sperm production in these testicles. By cryopreserving testicular tissue in these situations, there is a potential for preserving fertility, offering hope for future reproductive capabilities [104].

The cryopreservation of gonadal tissues, including testicular tissue, remains experimental; nonetheless, the optimization of testicular tissue cryopreservation procedures has gained attention due to the survival of germ cells in these tissues post-thawing [105], paving the way for the development of cryopreservation protocols now available for freezing testicular biopsies in both human and animal models [106].

#### 3.1.4. Sperm Cryopreservation and Xenografting

In general, sperm cryopreservation has become a frequent operating method in improving and propagating livestock genetic lines and species and assisting reproduction clinics in human therapies [79,107]. Regarding livestock management, the strategies for sperm cryopreservation are constantly advancing. In different studies, cryopreserved xenotransplants and germ cell transplantations have been performed as experimental models for preserving male fertility [108]. However, xenografting methods have been used with limited success for subsequent sperm extraction [109].

#### 3.1.5. Testicular Tissue Cryopreservation for Xenografting

In both human and non-human primates, xenotransplants and germ cell transplantation have been shown to induce spermatogenesis, significantly reducing the turnover time between generations [110]. Consequently, cryopreserved testicular tissue has the potential for indefinite storage [106,111]. Long-term storage has been verified in mice, and offspring have been obtained [112]. Moreover, spermatids were obtained from cryopreserved testicular tissue in farm animals, non-human primates, and humans in cases of non-obstructive azoospermia [113]. Performing testicular tissue xenotransplants is a valuable experimental procedure to study testicular physiology and germ cell development that may be useful for fertility preservation and reproductive medicine.

#### 3.1.6. Testicular Sperm Extraction (TESE)

Testicular sperm extraction (TESE) has been carried out successfully from fresh testicular tissue in human and non-human primates, obtaining viable offspring through IVF [114].

Considering the known resistance of ejaculated sperm to cryopreservation in mammals, future studies should directly compare this to the cryopreservation resistance of sperm obtained from immature testicular biopsies that mature ex situ (in vitro or after xenografting) in models like rodents or non-human primates. Such comparative research could unveil important insights into the cryopreservation resilience of different sperm sources.

#### 3.1.7. Testicular Sperm Extraction (TESE) and Xenografting

Some attempts to culture immature testicular tissue have been carried out during the past years; however, the results are variable, and procedures failed in different species, including men [115,116,117]. On the contrary, xenografting-derived immature testicular tissue has provided encouraging results in rodents. When donor testis material, obtained from immature rats on postnatal day 8, was cultured ex vivo for 4 days and xenotransplanted under the dorsal skin of castrated nude mice, complete spermatogenesis was achieved [118]. Remarkably, the viability of this immature testicular tissue was maintained, and it sustained long-term survival in vivo in the recipient mice. Furthermore, immature testis tissues from mice and a non-rodent species, rabbits, were cryopreserved, thawed, and then transplanted into mouse testes can also produce the restoration of spermatogenesis in both mouse and rabbit testicular pieces transplanted into mice [119]. Sperm from the transplanted testicular tissues generated mouse and rabbit offspring. Also, when implanting newborn hamster testicular tissue into castrated nude mice, grafts survived and maintained their size regardless of the recipient’s age [120]. At intratesticular and dorsal skin locations, non-human primate immature testicular tissue (marmosets) 6 months of age was xenotransplanted to 4-week-old immunodeficient Swiss Nu/Nu mice. After 4 to 9 months post-transplantation, complete spermatogenesis was seen in all recovered intratesticular transplants [121]. Cryomedia containing dimethyl sulfoxide (DMSO) showed better results when immature testicular tissue cryopreservation was performed in rhesus monkeys compared to ethylene glycol or low DMSO concentrations [122]. Moreover, the storage for 24 h on ice did not reduce the potential of immature rhesus monkey testes to survive as a xenograft and initiate spermatogenic differentiation [122].

#### 3.1.8. Microsurgical Epididymal Sperm Aspiration (MESA)

Microsurgical epididymal sperm aspiration (MESA) is a microsurgical option involving fine-gauge needle aspiration of epididymal sperm-containing fluid, and then high-power optical magnification microscopy is used to retrieve spermatozoa [123]. MESA and ICSI techniques offer new hope for many men to become biological fathers, yielding high fertilization rates [124,125,126]. The combination of both (MESA-ICSI) has been prescribed in those cases of bilateral congenital absence of the vas deferens and non-obstructive (severely impaired or non-existent sperm production) or obstructive epididymal cases (blockage in the male reproductive tract) [125,126,127]. Thus, MESA-ICSI can also be successfully performed using epididymal or testicular spermatozoa in patients with unreconstructable obstructive azoospermic infertility [125,126,127]. The technique has consistently achieved good results in excretory and secretory azoospermia cases that cannot be reconstructed by vasovasostomy or vasoepididymostomy [125,126]. The MESA technique has been used in different animal models, such as dogs [128] and rats [129]. Recently, a new approach for sperm retrieval for obstructive azoospermia cases called minimally invasive epididymal sperm aspiration (MIESA) has been developed using a tiny keyhole incision, and the epididymis is exposed without testicular delivery with low risk of complications [130]. MIESA is performed using loupe magnification rather than an operating microscope. Using MIESA, millions of motile sperm can be retrieved for cryopreservation [130].

### 3.2. Limitations and Challenges

#### 3.2.1. Genetic and Epigenetic Considerations: Risk of Transmission of Genetic Disorders

Genetic screening is a diagnostic and therapeutic search to detect individuals whose offspring are susceptible or at risk for genetic disease [131]. Prenatal molecular diagnosis with FISH or PCR technology can identify severe genetic disorders, permitting prompt treatment or the selective termination of pregnancy [132]. Therefore, carrier screening identifies individuals for serious recessive and harmful genes in a short period to reduce the incidence of genetic sex-linked diseases, translocations, recurrent miscarriages, and aneuploidies [133]. Using mice and rats as models, the successful fertilization achieved by injecting immature spermatozoa, spermatids, and secondary spermatocytes into oocytes has greatly expanded treatment options for severe male infertility. As the prevalence of andrological issues becomes a more common reason for resorting to ARTs, it becomes increasingly important to investigate the potential genetic and infectious disease risks associated with sperm-derived factors. The identification of male infertility is crucial because of the risk of the genetic factors involved being transmitted to the offspring. Genetic disorders can be identified in about 15% of male infertility cases [134]. Several ARTs, such as ICSI and IVF, are still surrounded by myriad problems that must be solved to optimize their results and reduce possible genetic imprinting disorders, the risk of zygotic loss, and low birth weight [135]. Epigenetic reprogramming is particularly susceptible to environmental conditions. Several conditions are caused by an imprinting defect in newborns associated with ARTs. Animal studies report disordered gene expression and epigenetic changes derived from environmental endocrine disruptors, oxidative stress, and methylation errors in imprinted genes following in vitro embryo culture [136]. The morphological and genetic characteristics of the sperm, the age of the gamete, and the hormonal balance seem to be essential factors [137]. Little evidence supports a correlation between increased paternal age and the incidence of chromosome anomalies. Although some studies demonstrated an increased risk of congenital abnormalities with advanced paternal age, such as unbalanced complements, inherited reciprocal translocations, and down syndrome, the incidence of severe non-chromosomal congenital disabilities, especially those arising from new autosomal mutations, also increases with paternal age due to impaired sperm function [138]. The experience gathered from various micromanipulation methods of gametes, including zona-opening procedures, subzonal sperm insertion, and sperm microinjection into the ooplasm, have demonstrated not only their clinical utility in assisted fertilization but also suggests the potential for selective application of these techniques and their combinations in the treatment of male infertility. The possible interference of gamete micromanipulation during ICSI with genomic imprinting and pre-zygotic sperm selection as potential teratogens could be important factors to consider [139]. ICSI produces chromosomal abnormalities, observed not only in infertile men and their spermatozoa but also in pre-implantation embryos resulting from microinjection [140]. Mutations of X-chromosomal or Y-chromosomal genes should play a significant role in male fertility disorders [141,142]. The source of sperm and the cause of the sperm defect appear not to affect the success of ICSI, whether the spermatozoon is fresh, frozen, ejaculated, epididymal, testicular, or from men with severe defects in spermatogenesis [143]. Even for men with oligospermia or azoospermia caused by obstruction or germinal failure, ICSI may be performed successfully [143]. Moreover, studies of ICSI pregnancies have revealed no increased risk of congenital malformation, autosomal abnormalities, and sex chromosomal abnormalities associated with azoospermia [144]. Apart from the transmission of genetic and chromosomal disorders and the increase in sex chromosomal aneuploidies associated with severe spermatogenic defects, the risk of serious adverse effects in offspring derived from ICSI, such as cardiac and genitourinary malformations, is comparable to natural conception [145].

Fertility preservation technologies have been significantly advanced through research on domestic animals. Thus, different animal species have been used as models for studying congenital abnormalities of genetic and environmental causes. Several commercial laboratories can perform genetic diagnostics for dogs and cats to obtain DNA test results related to mutations that cause health problems or alterations derived from reducing genetic variation, selection pressure, management in closed populations, and historical bloodlines [146]. Moreover, livestock models have made significant contributions to biomedical advancements, especially in the field of ARTs. For instance, several goat breeds are predisposed to malformations and metabolic diseases [147]. To detect mutant gene carriers in goats, it is crucial to avoid polygenic disorders (udder problems in females and gynecomastia in males) [148]. Other problems derived from gene carriers (pleiotropy) found in caprine species include intersexuality, early abortion, and arthritis [147]. In livestock, the overuse of popular sires is the most problematic breeding practice since it has also led to the dissemination of many inherited defects due to genetic diversity decline [149]. In bovine, the lower developmental capacity of in vitro-produced embryos could be due to the stress to which the gametes or embryos are exposed during in vitro embryo production, including hormone-supplemented medium, sperm handling, and cryopreservation [150]. ARTs can also be detrimental to farm animals. The potential adverse effects of IVF or ICSI on embryo development in bovine species could be attributed to disruptions in the physiological epigenetic profile of the gametes. This disruption might contribute, at least partially, to epigenetic disorders, leading to aberrant gene expression and chromosomal abnormalities in the embryos [151]. Most studies of genomic imprinting have focused on mouse models [30]; however, there is an increasing interest in new candidate genes and the phenotypic effects of imprinted genes in domestic livestock species such as cattle, sheep, and pigs [152].

#### 3.2.2. Sperm Quality-Related Issues and Fertility Treatment Constraints

MESA, TESE, and TESA techniques have been recommended for retrieving an adequate number of sperm for immediate use or cryopreservation purposes. Still, there is a lack of information on the quantity and quality of sperm retrieved [153]. Although performing testicular sperm extraction using these techniques has increased sperm retrieval rates, in vitro selection and processing of quality sperm plays an essential role in the success of future fertility; however, this practice has raised concerns because potentially genetically immature germ cells, that is, germ cells that have not completed epigenetic reprogramming may cause their infertility problems [154]. Testicular spermatozoa have been cryopreserved in secretory azoospermia cases for use later in ICSI or AI procedures [155]. However, in cases of non-obstructive azoospermia, such as in Klinefelter syndrome, the ICSI outcome was not affected by whether the retrieved epididymal or testicular sperm is fresh or frozen, neither by the technique of spermatic recovery [156]. Then, sperm cryopreservation may become an alternative to repeated surgery for obtaining testicular sperm for subsequent ICSI treatment [101].

Sperm cryopreservation should be considered in every surgical sperm retrieval case, including individuals with few testicular sperm collected [124]. Spermatids have been recovered from testis biopsies, but the fertilization and pregnancy rates using spermatids have been inefficient [157]. Additionally, sperm retrieval from testes followed by ICSI has also been employed as a resource in cases of severe oligozoospermia (sperm count < 5 × 10^6^ mL), necrozoospermia, and anejaculation by using multiple excisional testicular biopsies or less invasive alternatives such as percutaneous aspiration techniques to improve the rate of spermatozoa recovery [153].

Fertility preservation is crucial for individuals before treatments that can impair fertility [80]. Thus, sperm cryopreservation before oncological therapy starts is currently the only method to preserve future male fertility, and about 15% of men will use their cryopreserved sperm because of temporary or persistent azoospermia after the administration of gonadotoxic, chemotherapeutic, cytotoxic agents (chemotherapy) and radiation exposure (radiotherapy) in cancer treatment [80]. Prepubertal individuals cannot benefit from sperm cryopreservation because they only have spermatogonia and spermatocytes in their testes; however, testicular samples can be cryopreserved for future maturation treatments of immature germ cells [158].

In mammalian models such as dogs, the epididymal sperm has excellent potential for improving fertility. In case of death, orchiectomy, or even in azoospermic individuals, the epididymal sperm opens new horizons to generate progeny [128]. In canine, freeze-derived tolerance of epididymal sperm, that is, the ability of epididymal sperm to maintain quality and viability through the cryopreservation process, seems lower than that of ejaculated spermatozoa; however, epididymal sperm can be cryopreserved and used for AI successfully in dogs [128].

Xenotransplantation enhances the therapeutic effects of stem cell transplantation on azoospermic animal models. For example, analyses by type of azoospermia indicated that the use of stem cell transplantations in rat or mouse models had a therapeutic effect. Still, no effects were observed in azoospermic hamsters [159].

Hormonal regulation of spermatogenesis is well known in rats, monkeys, and human models, with slight differences among species. FSH seems decisive in the progression of type A to B spermatogonia and, in synergy with testosterone, in regulating germ cell viability [160]. Even in rats, testosterone is critical for the adhesion of round spermatids to Sertoli cells and promotes the release of mature elongated spermatids from the testis [160]. Treatment of non-human primates and men with steroidal contraceptives indicates that impairment of spermiation is a critical factor in azoospermia [160]. Therefore, spermatogenesis dysregulation can be caused by genetic factors. Several animal models with gene manipulations or deficiencies (mutations) exhibit various morphological and functional abnormalities that lead to infertility. In humans, several mutations related to infertility (oligozoospermia and globozoospermia) have been described [161]. The causative genes (including non-obstructive azoospermia), identified mutations, and mutation rate have been studied in knockout/mutated mouse models.

#### 3.2.3. Efficiency and Success Rate of Current Assisted Reproductive Technologies for Resolving Male Infertility

The effectiveness of current Assisted Reproductive Technologies (ARTs) in enhancing male fertility varies considerably. For example, it appears that ICSI of a single spermatozoon into the cytoplasm of a fertilizable metaphase II is much more efficient than subzonal insemination (SUZI) and partial zona dissection (PZD) for resolving cases of severely impaired semen quality or idiopathic infertility, while conventional IVF can be a successful procedure in tubal and unexplained infertility [162]. In male infertility due to unreconstructable obstructive azoospermia, microsurgical epididymal sperm aspiration can reach fertilization rates per metaphase II oocyte of 63%, and with testicular sperm aspiration, the fertilization rate was 59% [163]. After TESE, sperm retrieval was positive in 92% of cases with aspermia and 58% of patients with non-obstructive azoospermia [164]. When combining TESE-ICSI to treat non-obstructive azoospermia, a significantly lower proportion of embryos developed to the blastocyst stage than in cases with aspermia and those after ICSI with frozen-thawed ejaculated sperm (23% vs. 43% and 47%, respectively), [165].

In other cases, such as individuals with non-obstructive azoospermia, using testicular fine needle aspiration (TEFNA), a significantly lower yield was obtained compared to TESE (pregnancy rates 13 vs. 29%, respectively) [166]. However, TEFNA should be considered a choice whenever sperm recovery is attempted in non-obstructive azoospermia [167]. Other techniques, such as percutaneous testicular sperm aspiration (PESA), are suitable for collecting mature spermatozoa in many cases with non-obstructive azoospermia because it is safe, minimally invasive, and well tolerated [168]. Moreover, PESA is simple, efficient, and feasible for diagnosing azoospermia [169]. Fertilization rates were similar when using TESE-N, PESA, and TESE-P fertilization, ranging from 72% to 77% [170].

The efficiency of micro-dissection testicular sperm extraction (MicroTESE) in cases with non-obstructive azoospermia regarding fertilization rate was 58.4% [171]; however, specialized expertise can be potentially traumatic to the testicular tissue [172].

In cases of obstructive azoospermia, ICSI could be planned in conjunction with surgical sperm retrieval [173]. Using frozen sperm compared to fresh sperm in the conventional TESA-ICSI technique, the fertilization rates obtained were 71.4% and 73.4%, respectively [174]. TESA-ICSI is a relatively efficient and safe way in cases of temporary ejaculation failure (TEF) to obtain fertilization rates of 65.25% compared with those obtained in cases of obstructive azoospermia (66.43%) [175]. When using fresh and frozen-thawed epididymal spermatozoa, the fertilization rate was 56% versus 53% with ICSI [176]. The outcome of ICSI using fresh and frozen-thawed spermatozoa after retrieval by PESA was similar to that by MESA; therefore, the epididymal sperm cryopreservation in cases of obstructive azoospermia is feasible and efficient [176]. Thus, ICSI combined with epididymal or testicular sperm achieves high fertilization and pregnancy rates and constitutes an efficient alternative in treating cases suffering from testicular failure or azoospermia not amenable to surgical reconstruction [163].

Round spermatids have been used as substitute gametes in reproductive research and as a treatment for non-obstructive azoospermia [177]. The efficiency of fertilization and pregnancy is much lower after round spermatid injection (ROSI), with a fertilization rate of 38.7% compared to injection with mature sperm using ICSI of over 50% [178].

## 4. Advances in Male Fertility Preservation Techniques

The landscape of male fertility preservation has undergone a significant transformation, especially with the convergence of cutting-edge technologies and our expanding knowledge of animal models. This section explores the emerging field of male fertility preservation, underscoring the pivotal role of artificial gametes, stem cell-based therapies, in vitro techniques, and cloning. As we bridge the divide between animal models and human applications, these innovations present promising or addressing male fertility challenges.

### 4.1. Animal Studies and Implications for Human Fertility

Animal models have been instrumental in advancing the field of artificial gamete generation, offering invaluable insights with implications for human fertility treatments. While mice models have proven pivotal in establishing protocols to study male infertility, there remain significant challenges when translating these accomplishments into human applications. One of the predominant challenges arises from ethical and regulatory considerations. For instance, the use of human embryonic stem cells in research is often enveloped in ethical debates and stringent regulations [179].

Biologically, humans and mice present notable differences. Fine-tuning techniques for mice may not directly apply to humans due to variations in germ cell developmental timelines, among other factors. Additionally, technical challenges persist, and many developments remain experimental to date. For a summary of how various animal species contribute to male fertility preservation research, please refer to Table 3, which presents a comparative analysis of their roles in this field.

### 4.2. Artificial Gametes

Artificial gametes, often referred to as in vitro-derived gametes or synthetic gametes, represent a cutting-edge frontier in reproductive biology. These are sex cells—sperm or eggs—generated outside the human body, typically derived from germline stem cells. Once germ line cell types are obtained, they can be converted from the least differentiated state to the most differentiated. Their potential application in treating infertility and broadening reproductive options offers a transformative approach to reproductive medicine.

#### 4.2.1. Deriving Germ Line Cells from Pluripotent Stem Cells: Embryonic and Induced Pluripotent Stem Cells

In the intricate landscape of cell biology, pluripotent stem cells stand out for their exceptional ability to differentiate into virtually any cell type in the human body, including germ line cells. In creating artificial gametes, the potential to derive germ line cells from pluripotent stem cells represents a crucial advancement. It provides a practical approach to tackling reproductive challenges like infertility and contributes to understanding the basic mechanisms of human reproduction. Embryonic stem cells (ESCs) are pluripotent stem cells derived from the inner cell mass of a blastocyst, an early stage embryo. Due to their pluripotency, ESCs can differentiate into any of the three germ layers: endoderm, mesoderm, or ectoderm. This ability to transform into diverse specialized cell types makes them invaluable for research, therapeutic applications, and understanding early human development. The first establishment of mouse embryonic stem cells (ESCs) was achieved by two researchers independently: Martin Evans and Matthew Kaufman at the University of Cambridge in 1981 and Gail R. Martin at the University of California, San Francisco, also in 1981 [186,187].

Induced pluripotent stem (iPS) cells are a type of pluripotent stem cell that can be generated directly from adult cells, effectively bypassing the need for embryos. Professor Shinya Yamanaka and his team at Kyoto University pioneered the concept of iPS cells in 2006. They successfully reprogrammed mature mouse fibroblasts into pluripotent stem cells by introducing a combination of four specific genes, now famously known as the “Yamanaka factors”: Oct4, Sox2, Klf4, and c-Myc. These factors act as molecular switches that reset the adult cells into a pluripotent state, mirroring the properties of embryonic stem cells [190].

Embryonic stem cells (ESCs) and induced pluripotent stem cells (iPSCs) are promising for deriving germ line cells due to their pluripotent properties. Studies have shown that under specific culture conditions, both ESCs and iPSCs can differentiate, for instance, into primordial germ cell-like cells (PGCLCs) [205,206,207]. These cells can further develop into more mature germ cells, including spermatogonia, spermatocytes, and even spermatids in vitro. The induction of PGCLCs from pluripotent stem cells involves mimicking the signaling events that occur during early embryogenesis, typically involving the modulation of key signaling pathways such as BMP [207] and the expression of key germ cell genes. This ability to generate germ cells from pluripotent stem cells offers significant potential for studying the mechanisms of germ cell development and could potentially be used for therapeutic applications in infertility treatments.

#### 4.2.2. Mouse Models for Artificial Gametes Generation

Both Toyooka’s and Geijsen’s teams independently succeeded in generating primordial germ cells (PGCs) from embryonic stem (ES) cells. Toyooka’s group successfully achieved in vitro production of functional germ cells from embryonic stem (ES) cells [207]. They utilized knock-in ES cells where GFP or lacZ was expressed from the endogenous mouse vasa homolog (Mvh), a marker specifically expressed in differentiating germ cells. The approach used allowed them to visualize germ cell production during in vitro differentiation. The emergence of MVH-positive germ cells was contingent on embryoid body formation and was significantly enhanced by bone morphogenic protein 4-producing cells. When these ES-derived MVH-positive cells were transplanted into reconstituted testicular tubules, they participated in spermatogenesis, confirming that the ES cells could produce functional germ cells in a laboratory setting. They progressed further by deriving gonocytes from the PGCs and, subsequently, spermatogonial stem cells (SSCs) from these gonocytes.

On the other hand, Geijsen’s group produced spermatid-like cells, termed haploid-like (HL) cells, from embryonic stem cells [205]. They utilized embryonic stem cells in embryoid body systems. Since embryoid bodies sustain blood development, the researchers hypothesized that these structures might also support the formation of primordial germ cells, the founder population of gametes in mice. By isolating primordial germ cells from embryoid bodies, they were able to derive continuously growing lines of embryonic germ cells. These germ cells displayed erasure of methylation markers (imprints) characteristic of the germ lineage. They also demonstrated that embryoid bodies support the maturation of primordial germ cells into haploid-like male gametes. When these gametes were injected into oocytes, they restored the somatic diploid chromosome complement and developed into blastocysts, though live offspring were not achieved.

In a pivotal 2006 study, Nayernia et al. developed both SSCs and sperm cells from mouse ESCs [188]. By using ICSI with these artificially produced sperm, they generated viable embryos. Normal offspring were produced when these embryos were transferred to surrogate mothers, marking a significant milestone in the field. Further advancements were observed with induced pluripotent stem (iPS) cells. Hayashi’s team, 2011, produced epiblast cells from which they derived PGCs [208]. By 2012, Zhu’s team had generated both SSCs and HL cells from iPS cells [209]. Imamura’s study stood out as they first created iPS cells from hepatocytes and then successfully derived PGCs from these cells [206].

Ishikura et al. (2021) pioneered a methodology to systematically derive the entire spectrum of male germ cell types from pluripotent stem cells [210]. In their study, day 4 cultured mouse primordial germ cell-like cells (mPGCLCs) derived from mESCs were employed as starting materials to differentiate them into spermatogonium-like cells. These cells were then developed into germline stem cell-like cells (GSCLCs) that exhibited robust spermatogenesis both in vivo and in vitro. The study highlighted the significance of genome-wide DNA demethylation for proper spermatogonia differentiation. GSCLCs derived under these conditions showed a transcriptome and DNA methylome closely resembling genuine germline stem cells, though some differences persisted. These differences may impact the function of spermatogonia, but the derived GSCLCs still demonstrated efficient spermatogenesis and produced viable offspring through techniques like ICSI/ROSI. The study emphasizes that while the whole process of male germ-cell development has been reconstituted in vitro with the assistance of embryonic/neonatal testicular somatic cells, future challenges involve inducing specific stages of germ-cell development under defined conditions without relying on testicular somatic cells. The methods and approaches pioneered in this research offer insights into male germ-cell development, its challenges, and potential therapeutic solutions.

iPSCs vary in their states across species. Specifically, mouse iPSCs are predominantly in the naïve state, whereas human iPSCs tend to be in the primed state. Similarly, mouse ESCs are generally considered naïve in contrast to human ESCs, which are in the primed state [211]. These distinct states demand growth factors for self-renewal and can be transitioned between specific culture environments. This distinction is essential for understanding the developmental potential of these cells to derive human germ line cells.

#### 4.2.3. Artificial Gametes Generation in Humans

Several research groups have successfully produced primordial germ cells (PGCs) from human embryonic stem cells (ESCs), including teams led by Kee (2009), Bucay (2009), Tilgner (2008 and 2010), and Aflatoonian (2009) [212,213,214,215]. Notably, Aflatoonian (2009) and Kee (2009) advanced this work by achieving the production of haploid-like (HL) human cells from ESCs. Regarding induced pluripotent stem (iPS) cells, Eguizabal’s groundbreaking 2010 study generated PGCs, marking an important milestone in the field [216]. Panula et al. (2011) mirrored this achievement and took it a step further by producing HL human cells from iPS cells [217]. To date, no research group has been able to create human embryos or offspring using artificial male gametes. As we discover more about deriving germ cells from pluripotent cells, certain human embryonic stem cell lines, notably H1, H7, and H9, have emerged as instrumental tools [218]. H1 and H9, in particular, have been highlighted as commonly requested by the National Stem Cell Bank [218].

In the seminal year of 2004, Clark and colleagues paved the way with their observations on embryoid body (EB) differentiation, revealing that spontaneous differentiation of human embryonic stem cells (hESCs) was capable of giving rise to putative germ cells expressing a myriad of genes, including DAZL, DPPA3, DDX4, and SYCP3 [219].

In 2015, it was learned that the pre-inducing hPSCs to specific states, such as a distinct pluripotent state or a mesoderm-like state, before venturing into direct germ-cell differentiation was beneficial for generating germ cell line cells [220,221]. Deriving germ cells from embryoid bodies is the emerging strategy, as demonstrated by both these research groups. At the same time, Irie et al. utilized mouse embryonic fibroblasts (MEFs) followed by a priming culture on vitronectin/gelatin [221], and Sasaki et al. initiated their cultures on laminin, they primed on plasma fibronectin [220]. In their research, Sasaki et al. pinpointed specific culture conditions and signaling pathways conducive to the efficient induction of human germ cell-like cells from pluripotent stem cells (hPSCs). The cells they derived displayed characteristics that aligned with those of early germ cells. Irie et al. delved into the pivotal role of the SOX17 transcription factor in determining the fate of human primordial germ cells (PGCs). Their findings highlighted SOX17’s essentiality in guiding human PGC development, a marked contrast to the mouse model where BLIMP1 assumes a dominant function. This study shed light on the intricate molecular mechanisms orchestrating human PGC specification.

More recently, a meticulous comparison of the gene expression profile across five distinct human embryonic stem cell lines cultivated on varied matrices was conducted. This study revealed that after several passages on laminin 521, a more homogeneous expression pattern of key pluripotency markers, including POU5F1, NANOG, SOX2, and GDF3, was discernible [218].

To date, the methodology for assessing the functionality of human germ cells derived from hPSCs has primarily revolved around protein- and gene-expression analyses. Key protein markers, including DDX4, POU5F1, SYCP3, DAZL, cKIT, PRDM1, SSEA1, DPPA3, and acrosin, have been employed to identify various stages and types of germ cell differentiation [218]. However, unlike in animals where functionality is confirmed by observing changing DNA contents during meiosis and the production of viable offspring using in vitro generated gametes, a definitive gold standard methodology for humans remains elusive due to ethical constraints.

Single-cell RNA sequencing (RNA-seq) has advanced our comprehension of germ-cell development, shedding light on the maturation trajectory from prenatal stages to adulthood. Irie et al. and Sasaki et al. have employed RNA-seq to highlight the critical roles of genes like SOX17 and to compare hPGCLCs with other germ cells [220,221]. This technology has unveiled the intricacies of adult germ cells, including identifying multiple distinct populations [222]. Furthermore, it has emphasized the significance of the somatic environment in germ cell development, with recent studies underscoring the potential of three-dimensional culture conditions in aiding differentiation [223]. Through RNA-seq, researchers can better refine differentiation protocols and compare in vitro germ cells to their in vivo counterparts [218].

While these groundbreaking studies have advanced our understanding of human germ cell differentiation, further research is imperative to bridge the gap between these findings and the successful generation of fully functional human artificial gametes.

#### 4.2.4. Other Animal Models for Artificial Gametes Generation

Rat. The rat is a crucial biomedical research model, with its pluripotent stem cells offering unprecedented insights into reproductive medicine. Recent studies have highlighted the potential of rat-derived pluripotent stem cells in germline transmission and the production of viable offspring. Hamanaka’s pivotal study underscored the capability of riPSCs to contribute to germline transmission. Through the reprogramming of rat somatic cells using three key factors—Oct3/4, Klf4, and Sox2—riPSCs were derived, showing competence in contributing to both intraspecific rat and interspecific mouse-rat chimeras [193]. The next logical step was the generation of Functional germ cells from Pluripotent Stem Cells in Rats. Oikawa’s research aimed to derive functional primordial germ cell-like cells (PGCLCs) from rat pluripotent stem cells. Their findings demonstrated the successful induction of PGCLCs capable of producing functional spermatids, which subsequently sire viable offspring [224].

Iwatzuki et al. made significant strides in deriving and propagating post-implantation epiblast-derived pluripotent stem cells (rEpiSCs). Optimizing culture conditions, they revealed rEpiSCs’ potential to be reset to a naive pluripotent state using exogenous Klf4. Crucially, these rEpiSCs demonstrated competency in generating primordial germ cell-like cells, leading to functional gametogenesis and the birth of viable progeny Link to source.

Ming-Gui Jiang and colleagues presented a groundbreaking methodology optimizing induction media, notably with knock-out serum replacement and vitamin C. Their approach facilitated the efficient derivation of riPSCs from Dark Agouti rat fibroblasts and Sertoli cells. These riPSCs, exhibiting stable undifferentiated states over 30 passages, differentiated into various cell types, including germ cells. A notable achievement was the production of transgenic riPSCs using the PiggyBac transposon, setting the stage for transgenic rat creation via germ line transmission. Their success in obtaining transgenic offspring using the derived gametes positions riPSCs as a valuable tool for rat genetics and genomics, emphasizing their relevance in artificial gamete derivation [225].

In summary, these groundbreaking advances in rats echo similar accomplishments observed in mice, highlighting the potential of these phylogenetically related animal models in pioneering the fields of reproductive biology and genetic engineering.

Rabbits. In rabbits, primordial germ cell (PGC) specification happens at the posterior epiblast at the beginning of gastrulation, similar to the development in bilaminar discs observed in humans and most mammals, contrasting with rodent development as egg cylinders [226]. From newly derived rabbit pluripotent stem cells, rbPGC-like cells can be robustly and rapidly induced in vitro using WNT and BMP as morphogens, and therefore, SOX17 identified as the pivotal regulator of rbPGC fate, consistent with its role in several non-rodent mammals [226]. The study suggests that the development of bilaminar discs is a key factor determining PGC regulators, independent of the diverse development of extraembryonic tissues.

Pluripotent stem cell lines have been derived from rabbits [194]. These cell lines express stem cell-associated markers and maintain apparent pluripotency during multiple passages in vitro. However, their complete in vivo pluripotency has yet to be convincingly demonstrated. The difficulty in achieving fully pluripotent stem cell lines in rabbits, as compared to mice, is due mainly to suboptimal rabbit markers for embryonic stem cells (ESCs), which are not always specific to the pluripotent inner cell mass. Besides, efficient somatic cell reprogramming requires rabbit-specific pluripotency genes, which are currently challenging to identify and utilize. While germ line cell types have not yet been derived from rabbits, this animal model holds promise for artificial gamete generation to address human infertility issues. The rabbit model offers advantages, such as its physiological similarities to humans and a shorter reproductive cycle, making it a potential candidate for reproductive research.

### 4.3. Spermatogonial Stem Cell-Based Therapies

Spermatogonial stem cells (SSCs) are critical components of male fertility, maintaining a constant pool of cells in the testis and facilitating sperm production through spermatogenesis. Their unique properties lend them to promising animal reproduction and regenerative medicine applications. These applications include gene targeting, inducing pluripotency, and potentially restoring fertility. Techniques such as SSC transplantation and testis tissue xenografting have been developed, though these remain technically challenging in large animals and humans. Advancements in SSC culture methods have further expanded their potential use. In addition, the successful demonstration of in vitro spermatogenesis in mice offers exciting potential for addressing reproductive issues in the agricultural sector and human fertility treatments.

#### 4.3.1. SSCs Transplantation

The development and application of spermatogonial stem cell (SSC) transplantation can be traced back to the late 20th century. In 1994, a foundational study by Brinster and Zimmerman demonstrated that germ cells could colonize mouse testes and initiate spermatogenesis, setting the stage for subsequent investigations into spermatogonial stem cell (SSC) transplantation [184]. Two years later, in 1996, Brinster’s lab conducted a seminal experiment where they successfully transplanted frozen-thawed spermatogonial stem cells into the seminiferous tubules of recipient mice [227]. The ability of these cells to recolonize, differentiate, and commence spermatogenesis confirmed the stem cell properties of spermatogonial stem cells.

The early 2000s saw the technique expanded to larger animal models. Between 2002 and 2003, successful SSC transplantations were reported in goats, boars, and cattle [195,199,200]. These results suggested the broader applicability of the technique beyond small mammals. By 2013, another significant advancement occurred when SSC transplantation was successfully used to restore monkey fertility [228], highlighting the potential for its application in primates.

From 2012 onwards, research has been geared towards refining the SSC transplantation methodology. Focus areas include improving colonization and spermatogenesis efficiency and evaluating the associated risks. Additionally, there’s an ongoing exploration of the technique’s clinical application, particularly for fertility preservation in prepubertal boys undergoing treatments such as chemotherapy.

The development of SSC transplantation clearly illustrates the advances that have been made in this field over the past few decades. Still, it highlights the challenges before this procedure can be widely applied in clinical practice.

Spermatogonial stem cell (SSC) transplantation is a procedure that involves isolating SSCs from a donor testis and transplanting them into the testis of a recipient. The transplanted SSCs then colonize the seminiferous tubules and initiate spermatogenesis, thereby producing sperm carrying the genetic material of the donor. The potential of SSC transplantation for overcoming fertility problems is significant. SSC transplantation holds substantial possibilities for addressing fertility challenges based on several key applications. One primary application is in the realm of oncology. Men diagnosed with cancer often undergo treatments, such as chemotherapy or radiation therapy, which carry the risk of germ cell damage leading to infertility [201]. SSC transplantation offers a potential solution. Before commencing these treatments, SSCs could be harvested and cryopreserved. Post-treatment, these cells could be transplanted back, aiming to restore fertility.

Genetic anomalies are among the causes of male infertility. SSC transplantation introduces a therapeutic avenue whereby SSCs from an infertile patient are genetically modified ex vivo to rectify the inherent genetic defect. Once corrected, these cells could be reintroduced into the patient’s testes to potentially fix the infertility issue.

From a research perspective, SSC transplantation offers an unparalleled tool [229]. It enables the study of male germ cell development and spermatogenesis mechanisms. Understanding these processes in depth could pave the way for innovative treatments addressing male infertility. It’s worth noting that while these potential applications are promising, SSC transplantation is still mainly in the experimental stage. There are technical challenges to be overcome, such as the low efficiency of colonization of the transplanted SSCs and potential risks, such as the risk of transmission of diseases or abnormal cells. Therefore, more research is needed before this technique can be widely used in clinical practice.

#### 4.3.2. Animal Models Involving SSCs

Mouse. Spermatogonial stem cell (SSC) transplantation in mice involves the collection of SSCs from a donor mouse’s testes and their subsequent introduction into the seminiferous tubules of an infertile recipient mouse. To prepare the recipient, its native germ cells are typically eradicated using treatments such as busulfan or irradiation to create a niche for the incoming SSCs. Once the donor SSCs are isolated, they are injected into the rete testis of the recipient, from where they migrate to the seminiferous tubules. These transplanted SSCs then colonize the recipient’s testes, differentiate, and initiate the process of spermatogenesis, allowing the previously infertile mouse to produce sperm derived from the donor’s genetic material. Brinster et al. 1994 found that cells derived from the testis and transplanted into an infertile mouse testis can establish residence in seminiferous tubules and begin the process of spermatogenesis in over 18–37% of the recipient mice [184].

Farm animals. Spermatogonial stem cell (SSC) transplantation has also been explored extensively in farm animals as they hold great promise as animal models for infertility and in various aspects of livestock management and genetic improvement.

Approaches with primates. In 2012, researchers reported successfully using SSC transplantation to restore fertility in rhesus macaque monkeys [202]. Autologus spermatogonial stem cells were transplanted into monkeys rendered infertile due to chemotherapy. Following transplantation, embryos with donor paternal origin were produced. This study was significant because it demonstrated the potential of SSC transplantation in primates, bringing the technique closer to potential applications in humans.

### 4.4. Spermatogenesis In Vitro

Animal models have dramatically facilitated understanding the complexities of human male infertility, particularly the transformation of spermatogonial stem cells into mature spermatozoa. These models have been instrumental in in vitro spermatogenesis, a promising area with implications for both understanding and addressing human male infertility.

In a 2011 study by Sato et al., researchers successfully replicated the process of spermatogenesis in vitro using neonatal mouse testes. They produced viable sperm that resulted in healthy offspring and demonstrated the potential of cryopreserving these tissues for future applications, paving the way for further advancements in reproductive biology and medicine [189]. In the study on in vitro spermatogenesis, several key elements were pivotal in successfully reproducing this intricate biological process. Firstly, the researchers harnessed the potential of neonatal mouse testes, rich in gonocytes or primitive spermatogonia. These early stage germ cells provided an optimal starting point for initiating and sustaining spermatogenesis in an artificial environment. Secondly, choosing a serum-free culture media eliminated any inconsistencies that serum components might introduce, ensuring a controlled and supportive environment for the germ cells to thrive. Furthermore, the strategic positioning of the testes tissue fragments at the gas-liquid interphase was instrumental. This placement ensured optimal oxygenation, a critical cell differentiation and proliferation factor. Validating the efficacy of their methods, the researchers successfully used the in vitro-derived spermatids and sperm to produce healthy and reproductively competent offspring via ICSI. This demonstrated the functional quality of the produced germ cells and marked a significant achievement in the realm of reproductive biology. In humans, the process has been achieved up to the stage of elongated spermatids [230,231], but offspring have not yet been obtained. Meanwhile, in the domain of farm animals, the bovine model stands as a prominent example of advancements in this field.

The ideal in vitro spermatogenesis culture system would likely be a three-dimensional (3D) model that closely mimics the testicular microenvironment. This system should permit cells to interact within a matrix and with each other, thereby replicating natural tissue architecture. The culture method should incorporate both testicular somatic cells and germ cells, allowing for complete spermatogenesis. The system should not only support the development of spermatogonial cells, but also facilitate the progression of meiosis and ensure the functionality of haploid cells. The balance and composition of somatic cell populations would be crucial, and the incorporation of spermatogonia into reconstructed tubular structures should be efficient. Additionally, the ideal culture system should account for the role of hormones and other key molecules that promote spermatogenesis, such as those identified by Sanjo et al. in 2018 and 2020. Ensuring that these factors are present in the three-dimensional culture environment would likely enhance the process of spermatogenesis, leading to the production of functional spermatids.

### 4.5. Xenotransplants of Testicular Tissue

Testicular tissue xenografting is a technique wherein pieces of testicular tissue are implanted into immunocompromised mice, usually in the back subcutaneous tissue, facilitating the explant to grow and subsequently produce sperm. This groundbreaking method was introduced in 2002 when researchers successfully transplanted testicular tissue from immature pigs and goats into nude mice, evidencing its potential for fertility preservation [232]. The grafted tissue establishes a functional circulatory connection with the host mouse [233]. After forming this connection, a functional feedback loop is created between the mouse’s pituitary and the endocrine cells in the graft, leading to the growth of the xenografts and sperm production over time [183], sperm that even though did not undergo maturation in the epididymis, is still potent for fertilization when employed in intracytoplasmic sperm injection (ICSI) [183]. The technique’s efficacy has been demonstrated across various species, with sperm from the xenografts being utilized to initiate fertilization. In fact, the production of viable offspring has been reported in rabbits and pigs using this method [119,234,235] and even humans. In a groundbreaking study, cryopreserved prepubertal testicular tissues, when autologously grafted under the back skin or scrotal skin of castrated pubertal rhesus macaques, matured to produce functional sperm [236]. Not only did the grafts grow and produce testosterone over an 8- to 12-month observation period, but complete spermatogenesis was also achieved in all grafts. Remarkably, the sperm derived from these grafts could fertilize rhesus oocytes, resulting in preimplantation embryo development, pregnancy, and even the birth of a healthy female baby. This breakthrough suggests that testicular tissue grafting holds significant promise for preserving the fertility of prepubertal patients undergoing gonadotoxic therapies.

So far, testicular tissues from more than twenty mammalian species have undergone xenografting. Impressively, in most of these species, both the spermatogenic and steroidogenic functions of the testicular tissue are re-established in the grafts. The technique has shown potential, achieving complete spermatogenesis in the most species used [237,238]. Going one step further, researchers have successfully regenerated functional testicular tissue by grafting testicular cells in suspension isolated from neonatal porcine or rodent testes onto mouse hosts [239]. These transplanted cells autonomously reorganized both the spermatogenic and interstitial compartments of the testis, producing functional haploid germ cells. This groundbreaking discovery offers a novel in vivo system to study mammalian spermatogenesis and testicular morphogenesis, providing an accessible platform for further investigations into these processes.

### 4.6. Cryopreservation Techniques for Xenotransplantations

Animals have played an instrumental role in advancing the field of testis tissue cryopreservation. Through systematic cryo-banking of their reproductive tissues, researchers have refined techniques that enhance reproductive management with the unique contribution of varying cryopreservation methods tailored to species-specific needs. As an example of species-specific variations in cryopreservation requirements during slow freezing, mandrill and marmoset testicular tissues are effectively preserved using a medium with 10% DMSO and 80% FBS. In comparison, chimpanzee tissues only require 20% DMSO without FBS [240]. On the contrary, fast freezing can be a better option for wild ungulates to cause less damage to sperm cells recovered from testicular tissues [240].

Several cryopreservation protocols have been designed tailored to specific species, notably humans, monkeys, and other primates. A common element across these protocols is the use of cryoprotectants, with dimethyl sulfoxide (DMSO) [203,204] and ethylene glycol (EG), reference [203] being the most prevalent, albeit in varying concentrations. The precision in cooling and warming rates is consistently emphasized, given their essential role in successful tissue preservation. Culture media, like MEM or DMEM, often supplemented with fetal bovine serum (FBS) or other additives, are utilized in some protocols. Using bovine fetal serum (BFS) and other xenogeneic additives in the cryopreservation of human testicular tissue raises several considerations. Bovine fetal serum is a rich supplement that provides essential growth factors, proteins, and hormones that can support the viability and functionality of cells during the cryopreservation process. However, its use in human tissue preservation might introduce concerns about potential cross-species contaminants, immunogenic reactions, and the ethical implications of sourcing BFS [241].

Patra et al. (2021) discuss the methodologies employed in the cryopreservation of human testicular tissues [204]. Slow freezing emerges as a favored method, with two distinct approaches: uncontrolled and controlled. The former is cost-effective, requiring minimal equipment and cryogenic agents, and is time-efficient. However, controlled slow freezing is more prevalent due to its consistent outcomes. This approach, though, confronts challenges like varied cryobiological properties of cell types and the extracellular matrix in tissue fragments and potential cytotoxic effects from prolonged exposure to cryoprotective agents.

Conversely, rapid freezing, which minimizes cell dehydration, has been less successful. It often results in extensive cryoinjuries due to unpredictable intracellular ice formation, leading to a high rate of cell death. As a result, its application in preserving testicular cells and tissues is limited.

## 5. Future Perspectives: Animal Models in Next-Generation Reproductive Technologies

**Figure 2:**
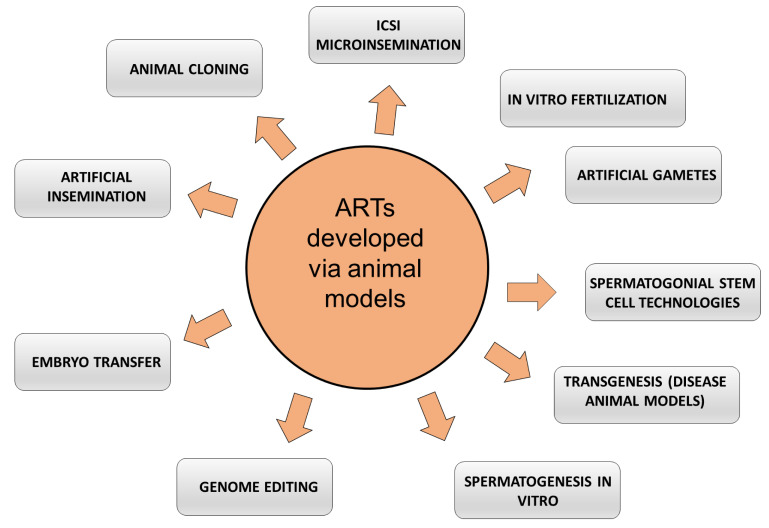
Overview of Technologies Derived from Animal Models for Human Male Fertility Treatment. This diagram illustrates key technological advancements, including AI, IVF, embryo transfer, transgenic/GM animal disease models, cloning, ICSI/microinsemination, artificial gametes, SSC technologies and transplantation, in vitro spermatogenesis, and genome editing, highlighting their potential applications and origins in animal research.

In the field of male fertility preservation, especially in scenarios involving cancer patients undergoing chemotherapy or radiation therapy, individuals facing surgeries that could impact fertility, and various other circumstances where fertility is potentially at risk, the landscape is evolving rapidly with the advent of emerging technologies. These innovative approaches shape the future perspectives in this field, heralding a new era where fertility preservation is becoming increasingly feasible and effective (Figure 2).

This section, therefore, aims to explore these advanced technologies, their mechanisms, potential applications, and the transformative impact they may have on preserving male fertility in challenging medical and personal situations. In the ever-evolving field of male infertility research, animal models continue to provide invaluable insights, laying the groundwork for innovative therapeutic strategies tailored for human application. Central to this progression is the use of CRISPR/Cas9 technology, which stands at the forefront of potential treatments for male fertility challenges. Simultaneously, the rise of OMIC technologies promises to reshape diagnostic paradigms, offering more timely detection of infertility. Complementing this, advancements in the targeted modification of the germ line genome can potentially provide personalized fertility solutions. As researchers delve deeper, mitochondrial replacement therapies are emerging as a beacon of hope for addressing infertility linked to sperm motility defects.

### 5.1. Advanced Bioengineering Techniques

Developing scaffolds using advanced biomaterials like alginate-based hydrogels, particularly in three-dimensional bioprinting, represents a significant step forward in creating stem cell systems in vitro. These scaffolds, designed to mimic the natural cellular microenvironment, offer the mechanical stability and biochemical properties necessary for the growth and differentiation of stem cells. As such, they hold tremendous potential in advancing in vitro gametogenesis, which is crucial for fertility preservation. Additionally, these techniques represent a significant advancement in animal welfare in scientific research. As scaffolds generated can mimic the structure and function of actual testicular tissue, they provide a viable alternative to using laboratory animals for various reproductive biology and toxicology studies.

This progress in biomaterial technology could be pivotal in overcoming current limitations and opening new avenues in reproductive medicine, especially in male fertility preservation. Alginate-based hydrogels, utilized as bio-ink, are distinguished by their biocompatibility, which is pivotal for the precise printing of elaborate three-dimensional structures. These bio-inks exhibit a harmonious balance in their mechanical attributes, integrating high strength and toughness with inherent biocompatibility, crucial for their effectiveness in 3D bioprinting applications [242]. Cell encapsulation is a process where cells are enclosed within a protective matrix or capsule, often made of biocompatible materials like hydrogels. This encapsulation mimics the natural cellular environment, providing the necessary support and signals for cell growth and differentiation. Cell encapsulation plays a pivotal role in creating in vitro stem cell systems in the context of male fertility preservation. Pioneering work in the field of alginates, a key material in this process, was conducted by E.C.C. Stanford in 1881. While exploring kelp for valuable products, Stanford discovered alginate and developed a method to extract a viscous substance, named ‘algin’, from algae, later precipitating it using mineral acid [243]. These technologies aim to develop tissue constructs with the requisite functional and biomechanical characteristics. An optimal structural and biochemical milieu is established by integrating materials such as poly (ethylene glycol)-diacrylate with sodium alginate in the hydrogel composition [242]. This exact biomaterial is crucial for encapsulating and precisely placing cells within the scaffolds.

While investigating the self-organization of testicular cells, Gao et al., 2020, found that when mouse testicular cells were encapsulated within the biomaterial Matrigel, they could self-organize into seminiferous tubules, complete with blood–testis barrier (BTB) formation and Leydig cell differentiation. This discovery is a significant advance, provides deeper insights into the functional role of the extracellular matrix (ECM) in testicular cell organization, and offers potential methodologies for reconstructing testicular tissue [191]. Their experimentation with different encapsulation methods, mainly using a combination of sodium alginate and collagen, further emphasized the ability to mimic natural testicular structures in vitro, a crucial step in fertility preservation and reproductive health research.

In the future, advanced testis culture systems based on microfluidic systems [244] will be developed, providing environments that closely mimic the physiological conditions of the testis. Microfluidic in vitro systems are miniaturized devices that manipulate small volumes of fluids, typically in the microliter or nanoliter range, through channels that are often only a few micrometers wide. These systems allow for the precise control and analysis of biological and chemical processes at a scale closely resembling the physiological conditions within living organisms.

### 5.2. Testis Organoids

Testis organoids represent a groundbreaking development in reproductive biology and medicine, offering a transformative approach to understanding and treating male infertility. Besides, organoids are another way to reduce the use of laboratory animals. These three-dimensional structures, created in vitro, mimic the architectural and functional aspects of the testicular environment. They provide an invaluable platform for studying the intricate processes of spermatogenesis, the maturation of sperm cells, and testicular physiology under controlled laboratory conditions. The advent of testis organoids opens up new possibilities for researching male reproductive disorders, testing the effects of drugs and environmental factors on male fertility, and exploring novel fertility preservation and restoration techniques. In their 2020 study, Maxwell and Woodruff made significant strides in understanding testicular organoid models, which are crucial for studying testicular physiology and spermatogenesis [245]. They conducted experiments to determine the influence of the culture microenvironment on the self-assembly of testicular organoids using immature murine testicular cells. Their research revealed that de novo tissues could self-assemble in various environments. Organoid assembly was age-dependent and more effective with immature cells than with pubertal or adult cells. Notably, they observed that immature cells could facilitate the building of organoids from adult cells in age-chimeric cell mixtures. The organoids formed in their study exhibited tubule-like structures and maintained key functions, such as testosterone and inhibin B secretion, responding to gonadotropins over 12 weeks. Considerable progress is being achieved in the evolving understanding of testis organoids [246,247]. Looking towards the future, two specific areas where these advances will be particularly impactful relate directly to testicular health and function. Firstly, applying testis organoids in drug testing and toxicology will become increasingly vital, for instance, to assess the effects of various substances on testicular tissue, thereby ensuring the safety of pharmaceuticals and identifying potential environmental hazards that may affect male reproductive health. Secondly, the potential of testis organoids in disease modeling is enormous. These organoids provide a unique opportunity to study the progression of testicular diseases, including various forms of testicular cancer, in a controlled and detailed manner.

### 5.3. Autologous Grafting of Immature Testicular Tissue

In the future, the field of male fertility preservation, particularly concerning autologous grafting of immature testicular tissue (ITT), will likely shift towards spermatogonial stem cell (SSC) transplantation as a more promising approach, primarily due to the potential of SSCs to be screened at the single-cell level using advanced omics techniques [248,249], allowing for a more detailed assessment and selection of healthy cells. Additionally, the isolation of SSCs presents an advantage in potentially separating them from any remaining cancer cells, thereby reducing the risk of cancer recurrence upon transplantation. Techniques for the isolation and in vitro expansion of spermatogonial stem cells have already been established in animal models such as mice [192] and bulls [197], demonstrating the feasibility of this approach. The application of these techniques in humans could revolutionize the process of fertility restoration, offering a safer and more effective method for young cancer survivors and others at risk of infertility. As research progresses, translating these techniques from animal models to human clinical practice could open new avenues for restoring fertility using SSC transplantation. Recently, significant advancements have been made in the field of reproductive biology, with the development of techniques to produce spermatogonial stem cells (SSCs) from human induced pluripotent stem (iPS) cells and expand them in vitro [250]. Importantly, this advancement could circumvent the need for extracting testicular tissue before cancer treatment, a process that is often invasive and can be challenging, particularly for young patients. This new approach offers a less invasive and potentially more efficient method for preserving fertility in individuals undergoing treatments that risk damaging their reproductive capabilities.

### 5.4. Transgenic and Genetically Modified Animal Models and Human Infertility

Various strategies employ spermatogonial stem cells (SSCs) for different purposes [251]. One primary application is fertility preservation, which aims to restore fertility in cancer patients undergoing treatments causing germ cell damage. In cell culture, the focus is on ensuring the survival, expansion, and potential genetic modifications of SSCs. Within this context, germ stem cells (GSCs) are a subset of SSCs that can self-renew and proliferate in vitro. Occasionally, multipotent germ stem cells (mGSCs) emerge from the SSC population during culturing, displaying characteristics of pluripotency [252].

Spermatogonial stem cells (SSCs) offer a more direct approach to transgenesis compared to the well-established embryonic stem (ES) cell-based techniques, especially in species like farm animals where standard ES technologies face challenges [251]. For ES cells’ genetic modification, constructs are injected into blastocysts with uncertain germline integration, while SSCs are inherently part of the germline, ensuring more predictable outcomes. Additionally, SSCs possess genetic and epigenetic stability, maintaining commitment to the germline phenotype and resisting differentiation into other cell lineages, which makes them a potentially safer option for gene therapies than pluripotent stem cells. This distinction is crucial in animals that produce fewer oocytes, have extended times to reach sexual maturity, or in species where ES technologies are inapplicable. SSC-based transgenesis methods, especially in farm animals, streamline the process by directly targeting and modifying SSCs, followed by their transplantation into recipient testes.

In the future, human transgenesis, which involves the genetic modification of human cells or organisms, will continue to be banned or heavily regulated in most countries due to ethical, legal, and safety concerns. However, animal transgenesis will persist as an instrumental approach in generating models for human fertility diseases. This method will allow for the thorough investigation of genes or genetic interactions that lead to infertility. Specifically, spermatogonial stem cell (SSC)-based genetic modification will be increasingly utilized in animal models biologically closer to humans than mice, such as pigs. These animals will offer a more relevant physiological context for studying human conditions despite the lack of an extensive range of embryonic stem cell lines compared to mice. Through SSC-based approaches in these alternative animal models, researchers will gain valuable insights into the genetic underpinnings of human fertility and reproductive health, advancing our understanding and treatment options for infertility.

### 5.5. Gene Editing

Genome editing technologies offer transformative possibilities for treating human diseases, including infertility. However, before clinical application, the long-term safety and potential immune reactions to these therapies need a thorough evaluation. Small animal models, like mice and rats, have limitations in accurately predicting long-term effects or secondary complications that may surface years post-treatment. Moreover, immunodeficient mouse models cannot provide insights into the host’s immune response to newly edited genes. In contrast, large animal models, such as dogs, pigs, and non-human primates, are more analogous to humans regarding anatomy, immune system, and lifespan [253]. They offer the advantage of longer study durations with more clinically relevant dosages for human scenarios. Using farm animals for genome editing studies allows for extensive blood and tissue sampling, facilitating a more profound understanding, especially of rare cell subsets. Thus, leveraging large animal models can be invaluable for advancing genome editing therapies, especially in human diseases like infertility.

### 5.6. Animal Models for In Vitro Spermatogenesis

In the future, animal models for in vitro spermatogenesis will continue to be instrumental in advancing our understanding of human infertility. These models will offer valuable insights into gamete development and maturation processes, enabling researchers to unravel the mechanisms and disruptions leading to infertility. The methodologies and insights gained from these studies will allow the development of therapeutic interventions to preserve human infertility. In the future, in vitro spermatogenesis in animals other than mice will be achieved. The biggest goal in this area would be to develop human in vitro spermatogenesis, a major breakthrough, offering new ways to understand and treat human infertility. Combined with technological advances, like high-throughput sequencing, spermatogenesis in vitro will allow us to obtain more details into the genetics and epigenetics studies of regulatory networks governing spermatogenesis. Moreover, developing in vitro culture systems based on a range of animal models will enable the dissection of signaling pathways and hormonal cues in spermatogenesis and the study of environmental, toxic, and genetic impacts on male fertility.

### 5.7. Challenges and Opportunities

Integrating technology with animal models in male infertility research has facilitated significant progress and introduced specific challenges. Ethical concerns arise, particularly with technologies such as CRISPR/Cas9 and germline genome modifications. It is essential to establish stringent ethical guidelines for these technologies. Additionally, the translational potential of findings from animal models to humans is challenging due to biological differences, necessitating continuous validation for human applicability. On the other hand, the confluence of these challenges also presents many opportunities. The very act of navigating the ethical landscape can lead to the establishment of global standards, promoting collaboration and consensus in the scientific community. The continuous refinement of animal models, driven by the need for better human analogs, can lead to more accurate and reliable research outcomes.

Moreover, as we delve deeper into the intricacies of male infertility, we can uncover previously unknown facets of reproductive biology, potentially leading to breakthroughs in other areas of medicine. Furthermore, incorporating emerging technologies like AI and machine learning with current research methodologies could revolutionize data analysis, paving the way for predictive modeling and personalized treatment plans. In essence, while the road ahead is fraught with challenges, these very challenges hold the key to unlocking unprecedented opportunities in the fight against male infertility.

## 6. Conclusions

In reproductive medicine, animal models have consistently proven invaluable tools, illuminating our understanding of male infertility and driving the development of next-generation reproductive technologies. As we stand on the cusp of a new era marked by groundbreaking technologies like CRISPR/Cas9 and advanced OMIC technologies, the role of these models becomes even more pivotal. They offer a lens to study intricate physiological processes and provide a platform to evaluate the efficacy and safety of novel interventions, bridging the gap between benchside innovations and bedside applications. However, as we forge ahead, it is imperative to approach this journey with a blend of optimism and caution. While the opportunities are vast, so are the challenges, particularly in ensuring these technologies’ ethical and responsible application. Our collective responsibility as a scientific community is to ensure that the knowledge gleaned from animal models translates into therapeutic benefits for patients without compromising ethical standards. The future of male fertility preservation hinges on this delicate balance. As researchers, clinicians, and stakeholders, we must navigate this path with integrity, foresight, and collaboration.

## Figures and Tables

**Table 1:** Classification of Factors Affecting Male Infertility.

Pre-Testicular Factors	
Hormonal Imbalances	These imbalances are characterized by the deficiency of hormones essential for sperm production, resulting in disorders like hypogonadotropic hypogonadism [2].
Systemic Illnesses	Systemic illnesses that may disturb male fertility include chronic diseases such as kidney failure, liver cirrhosis, and celiac disease.
Medications	Medications and drugs that may affect male fertility include treatments like chemotherapy, specific antifungal and antibiotic medications, testosterone replacement therapy
**Testicular Factors**	
Primary Testicular Failure	In this condition, the testes cannot produce sufficient quantities of healthy sperm. This can be due to radiation, trauma, genetics, or unknown reasons. Primary testicular failure can lead to endocrine dysfunction, resulting in a lack of testosterone or exocrine dysfunction that hampers spermatogenesis, ultimately contributing to male infertility [3].
Varicocele	This is a condition in which the veins on a man’s testicle(s) are too large, causing them to overheat and potentially affect sperm count and morphology. Varicoceles occur in around 15% to 20% of all males but are found in about 40% of infertile males [4].
Orchitis	This is an inflammation of the testicles that can be caused by infections
Cryptorchidism	This disorder is a prevalent male genitalia abnormality where one or both testicles do not descend into the scrotum, impacting 2–4% of male newborns [5].
Genetic Disorders	Genetic anomalies are detected in 10% to 20% of patients exhibiting severe disorders of spermatogenesis, including non-obstructive azoospermia and others like Klinefelter syndrome, Y-chromosome deletions, XX male, azoospermic factor (AZF) deletions, and congenital bilateral absence of vas deferens [6].
Testicular Cancer	While it is relatively rare compared to other cancers, it is the most common cancer in young men, especially those between the ages of 15 and 35 [7]. The development of malignant cells within the testicles. Indirectly, treatment, which may include surgery, radiation, or chemotherapy, can impact fertility. Even the presence of the cancer itself can affect sperm production and function.
Testicular Trauma	Direct physical injury to the testicles. Such injuries can be painful and may result from accidents, sports-related incidents, or direct blows. Temperature from trauma inflammation can raise the testicular temperature to levels incompatible with normal spermatogenesis and fertility [8,9].
**Post-Testicular Factors**	
Obstructive Causes	Blockages in the ducts that carry sperm from the testicles to the urethra, such as in the congenital bilateral absence of the vas deferens (CBAVD) [10].
Infections	Prostatitis, epididymitis, or sexually transmitted infections can lead to infertility. About half of infertility cases are attributed to male factors, with genitourinary tract infections accounting for roughly 15% of these instances [11].
Retrograde Ejaculation	When semen enters the bladder instead of emerging through the penis during orgasm [12,13].
Ejaculatory Duct Obstruction	This is a rare obstruction of the ejaculatory ducts (EDO) [14].
**Immunological Factors**	
Immunological Factors	Anti-sperm Antibodies: Some men produce antibodies that attack their own sperm, harming their fertility [1].
**Environmental and Lifestyle Factors**	
Endocrine Disruptors	These chemicals in the environment can mimic hormonal effects and disrupt the endocrine system once in the body. Endocrine disruptors have only recently emerged as a significant concern for causing male infertility. Exposures to chemicals, particularly bisphenol A (BPA), phthalates, and various pesticides, are being increasingly scrutinized for their potential adverse effects on male fertility [15].
Lifestyle Choices	Tobacco and alcohol consumption, drug use, obesity, stress, and malnutrition [16].
Occupational Exposures	Prolonged exposure to heat, heavy metals, radiation, or chemicals [17,18].
Electromagnetic Radiation	Extended exposure to electromagnetic radiation, commonly emitted by devices such as cell phones and monitors, has been increasingly linked to male infertility. Studies have highlighted its adverse effects on sperm parameters, including morphology, motility, and viability. A significant concern is the generation of reactive oxygen species (ROS) upon radiation exposure, leading to oxidative stress. ROS generation not only disrupts the redox equilibrium but also impairs sperm function and morphology, underscoring the potential risks of radiation on male reproductive health [19].
**Idiopathic Male Infertility**	
Idiopathic Male Infertility	A condition contributing to approximately 30–40% of male factor infertility, which is marked by a decrease in semen quality without an identifiable cause, often associated with oxidative stress [20].

**Table 2:** Overview of the Role of Animal Models in Reproductive Biology and Male Fertility Preservation.

Animal Model Examples	Classification	Contributions	Advantages	Limitations
*C. elegans*	Nematodes	Understanding Basic Reproductive Processes	Inexpensive; easy to maintain; Simple organism; fast breeding cycles; transparent body for easy observation	Far from humans on the phylogenetic scale
Drosophila	Insects	Understanding Basic Reproductive Processes	Inexpensive, easy to maintain	
Zebrafish	Fish	Understanding Basic Reproductive Processes	Inexpensive, easy to maintain; transparent embryos for real-time observation; genetic similarity to humans for certain pathways	Far from humans on the phylogenetic scale; external fertilization in zebrafish contrasts with internal fertilization in humans
Xenopus	Amphibians	Understanding Basic Reproductive Processes	Inexpensive, easy to maintain; large oocytes	Far from humans on the phylogenetic scale; amphibian reproductive system and development stages differ markedly from those in mammals.
Mice	Mammalian, Rodent	Development and refinement of fertility preservation interventions [21,22]; Initial Development and Validation of Reproductive Technologies (e.g., ICSI, IVF); genetic studies in fertility (Gene Knockout/Overexpression) [23,24]; Research on environmental and lifestyle impact on fertility [25]; Testing emerging technologies (e.g., mitochondrial replacement therapy [26,27,28], gene editing [29])	Easy to maintain, fast breeding cycles [30], many models of disease available; intermediate distance from humans on the phylogenetic scale	Limited representation of human biology and reproduction
Rats	Mammalian, Rodent	Development and refinement of fertility preservation interventions; Research on Environmental and Lifestyle Impact on Fertility	Easy to maintain, fast breeding cycles [30]; closer to humans on the phylogenetic scale than mice	Not many disease models are available, and ARTs not developed as in mice
Hamsters	Mammalian, Rodent	Development and refinement of fertility preservation interventions [31]	Easy to maintain, fast breeding cycles	Not many disease models are available, and ARTs not developed as in mice
Rabbits	Mammals	Development and refinement of fertility preservation Interventions; Research on environmental and lifestyle impact on fertility	Close to humans on the phylogenetic scale, the reproductive system is more similar to humans than smaller mammals like mice or rats [32]; spontaneous ovulation makes the model valuable for studies on ovulation and fertility treatments.	More extended gestation period compared to rodents (e.g., rabbit 30 days vs. mouse 20 days); Larger size and higher maintenance needs than smaller lab animals
Non-Human Primates	Mammals	Training in Reproductive Procedures	Very close to humans on the phylogenetic scale, therefore, closest physiological and genetic similarity to humans [33,34]	Expensive and complex maintenance; ethical and regulatory obstacles; access can be limited due to conservation concerns; Longer lifespan and reproductive cycle, which make developmental studies more difficult.

**Table 3:** Comparative Roles of Different Animal Species in Male Fertility Preservation Research.

Animal Model	Application in Fertility Preservation or Restoration
Mouse	Embryo cryopreservation [60]; sperm cryopreservation [180,181]; testis xenotransplantation from humans [182] and several species, like pigs and goats [183]; germ cell transplantation [159,184]; ROSI [177,185]; First embryonic stem cells (ESCs) isolation [186,187]; artificial gamete derivation up to offspring [188]; Spermatogenesis in vitro [189]; iPS cell derivation [190]; Alginate encapsulation [191]; in vitro expansion of SSCs [192]
Rat	germ cell transplantation [159]; MESA [129]; iPS cell derivation [193]
Hamster	IVF [59]; ICSI [31]
Rabbit	AI first experiences [36]; Sperm cryopreservation [180]; ET/IVF first experiences [48]; ES cell derivation [194]
Cats	Sperm cryopreservation [180]
Dog	AI first experiences [36]; Sperm cryopreservation [180]; MESA [128]
Pigs	Germ Cell Transplantation [195]; Sperm cryopreservation (high difficulty) [72]
Horse	AI first experiences [36]; Sperm cryopreservation [180]; ICSI [196]
Bulls	Sperm cryopreservation [47,180]; sexed semen [41]; ET [48]; in vitro expansion of SSCs [197]; SSC cryopreservation [198]; SSC transplantation [199]
Sheep	Sperm cryopreservation [180]; ET [49]
Goats	Sperm cryopreservation [180]; ET [49]; SSC transplantation [200]
Primates	Sperm cryopreservation [97]; TESE [114]; ROSI [177]; SSC transplantation [201,202]; Sperm cryopreservation [203,204]

ROSI = Round Spermatid Injection; TESE = Testicular Sperm Extraction; MESA = Microsurgical Epididymal Sperm Aspiration; ES cells = Embryonic Stem cells; IVF = In vitro Fertilization; ICSI = Intracytoplasmic Sperm Injection; AI = Artificial Insemination; ET = Embryo Transfer; SSC = Spermatogonial Stem Cells.

## Data Availability

Not applicable.
